# Supplementary material for: The MLL3/4 complexes and MiDAC co-regulate H4K20ac to control a specific gene expression program
Source: Life Sci Alliance. 2022 Jul 12;5(11):e202201572. doi: 10.26508/lsa.202201572 (PMC9275676; doi:10.26508/lsa.202201572)
Supplement: Supplementary file 2 [file LSA-2022-01572_TableS2.docx]

**Table S2. CRISRPR/Cas9 primers and editing construct sequences.**

| **Name** | **Sequence (5’ to 3’)** |
| --- | --- |
| sgRNA Spacers | |
| MLL3/KMT2C | |
| MLL3 sgRNA spacer  (MS599.KMT2C.g2.mut*) | UAUCUGUUGAACCAAAAAAAA |
| MLL4/KMT2D | |
| MLL4 sgRNA spacer 1  (SM124.KMT2D.g4) | AGGGGACUGAUAUGCACCGG |
| MLL4 sgRNA spacer 2  (SM125.KMT2D.g3) | UGCAUGGUCGGCAGGCGUAU |
| ELMSAN1 and TRERF1 | |
| ELMSAN1 sgRNA spacer (SM109.hELMSAN1.g1) | UGGCCUCAACUACCAAGUG |
| TRERF1 sgRNA spacer (CAGE654.TRERF1.g2) | GGCCAACCCAAAGGAGCGUU |
|  |  |
| Primers for PCR and Deep Sequencing Validation | |
| MLL3/KMT2C | |
| MLL3 primer 1  (PC865.F) | ACCTTGAGGGGGTTGAAGTCAAAGACT |
| MLL3 primer 2  (PC865.R) | CCACCTTAGAATTTGGAGACAGTACTTCCG |
| MLL4/KMT2D | |
| Deletion PCR primer 1 (SM124.DS.F) | GTCTTCCTGGGGCCTCGGCATTTGC |
| Deletion PCR primer 2 (SM125.DS.R) | TGGTCCTTCTCATTCCAACCTGACTCT |
| Inside deletion PCR primer 1 (SM124.Inside.F) | GGTGAGCTGTCAATCCTGCT |
| Inside deletion PCR primer 2 (SM124.Inside.R) | CAACCCACTCTCCTTCCTGC |
| 5’ of deletion PCR primer 1 (SM124.DS.F) | GTCTTCCTGGGGCCTCGGCATTTGC |
| 5’ of deletion PCR primer 2 (SM124.DS.R) | GCGGTAGGCCCATAAAAGCAGAACCA |
| 3’ of deletion PCR primer 1 (SM125.DS.F) | AGTGAGGAGAATGGGGCAGGAAATGG |
| 3’ of deletion PCR primer 2 (SM125.DS.R) | TGGTCCTTCTCATTCCAACCTGACTCT |
| ELMSAN1 and TRERF1 | |
| ELMSAN1 primer 1  SM109.hELMSAN1.DS.F | ATTGCACTTCTGTTCCCATCTTG |
| ELMSAN1 primer 2  SM109.hELMSAN1.DS.R | CGATGACTGATGGCTTGTGC |
| TRERF1 primer 1  CAGE654.TRERF1.F | GGCACTGGGGTGATACAAGTGGTGCC |
| TRERF1 primer 2  CAGE654.TRERF1.R | ACGGCAGCAAAGCAAAGCAAGCAGC |
